# Exploring the foundations of a digital health information service for patients with inflammatory bowel disease: a mixed method study in Gravitate-Health

**DOI:** 10.1186/s12876-024-03272-1

**Published:** 2024-05-24

**Authors:** Sigurd Maurud, Lene Lunde, Anne Moen, Randi Opheim

**Affiliations:** 1 Department of Public Health Science, Institute of Health and Society, Faculty of Medicine, University of Oslo, Oslo, Norway; 2 Department of Gastroenterology, Oslo University Hospital, P.O. Box 1089, Blindern, Oslo, 0318 Norway

**Keywords:** Health information, eHealth, Inflammatory bowel disease

## Abstract

**Background:**

Providing relevant digital health information of high quality may promote treatment adherence and self-management for patients with inflammatory bowel disease. The development of digital health services is optimised by considering end users’ needs.

**Aim:**

To identify key aspects required for digital promotion of inflammatory bowel disease patients’ self-management by exploring their health information needs and the preferences of both patients and healthcare professionals in relation to the digital provision of inflammatory bowel disease health services.

**Methods:**

Data from an audit of 1,481 electronic health record summaries from an inflammatory bowel disease help line, 17 semi-structured interviews with inflammatory bowel disease patients and 2 focus group interviews with 11 healthcare professionals were analysed.

**Results:**

Patients primarily contacted the hospital due to concerns about symptoms, examinations and tests, and medicines. Their concerns appeared to vary according to diagnosis, gender, age and disease duration. The interviews identified two overarching themes: (1) the available health information and patients’ health information needs, and (2) whishes, thoughts and preferences for a digital solution in IBD care with relevant and individualised information.

**Conclusions:**

The findings delineate key aspects for developing a suitable digital health information service. Patients seek information from healthcare professionals about treatment; however, in a digital solution, they want access to relevant and practical information about the disease, treatment and self-management. Both patients and healthcare professionals saw opportunities for increasing health data availability to patients. However, healthcare professionals expressed concerns about adapting, maintaining and ensuring the relevance of patient health information without increasing their workload and, thus, reducing quality of care.

**Supplementary Information:**

The online version contains supplementary material available at 10.1186/s12876-024-03272-1.

## Background

Inflammatory bowel disease (IBD), mainly represented by the diagnoses ulcerative colitis (UC) and Crohn’s disease (CD), is a chronic disease without a known aetiology [1]. Living with IBD can affect patients’ interpersonal relationships and leisure activities [2], can cause social isolation [3] and can negatively affect educational and professional performance [4], as symptoms can be present even when the disease is in remission [3, 4]. Many of the medicines used in IBD treatment—such as biologics, immunomodulators and corticosteroids—require good patient understanding, as they may be associated with significant risks [5]. Further, surgical procedures are often associated with a reduced quality of personal and social life [6, 7]. In terms of reported non-adherence to medical treatment rates in IBD, there is great variation, spanning from 7 to 72%, although with a higher frequency of reported non-adherence rates between 30% and 45% [8].

*Self-management* is a concept with a distinction that often is blurred along with another closely related concept, *self-care* [9]. The World Health Organization (WHO) has defined self-care as the ability to promote and maintain health, prevent disease and cope with disability and illness [10]. Self-management refers more specifically to patients’ ability to deal with the various physical, practical and psychosocial aspects of living with a chronic condition [11], and it is essential for controlling a chronic disease such as IBD [12, 13]. Self-management, however, involves complex activities and considerations; patients must self-monitor and manage symptoms, such as pain, urgency, fatigue [14], diarrhoea and weight loss [15], and practice health-promoting activities related to dietary intake, physical activity and complex medication regimens [16]. Good communication with healthcare professionals (HCPs) may facilitate patients’ uptake of essential health information [5, 17]. Knowledge about and experience with the disease may also optimise self-management [18, 19]. As such, education combined with digital approaches may improve treatment adherence among IBD patients [20]. Thus, digital health information interventions hold promising opportunities for self-management in IBD [21–24]. However, considering the great variation in patients’ expectations of digital health services across different chronic conditions and their unique manageability, treatments and symptoms [25], determining what constitutes an accessible, convenient and usable system largely depends on the end users’ needs and preferences [26].

IBD patients’ use of health information seems to vary based on characteristics such as gender, age and disease duration; for example, women and young patients appear to be more oriented towards information concerning daily self-management [27]. The time at which an IBD diagnosis is confirmed has been reported to be a critical time for receiving health information [28, 29], mainly general IBD information [27]. Patients seem to look more frequently for information about therapy and its efficacy during periods of active disease [30], while research and development appear to be of greater interest during remission [27]. UC patients have previously expressed a greater concern about their social lives, while CD patients have appeared to be more worried about heredity, surgery and risks associated with smoking [27].

Patients with IBD have been reported to receive limited information about long-term prognosis, possible complications, symptom management [31, 32], diet [28, 32], fertility, heredity, self-management [31], medications’ possible side effects [32] and adverse events [33]. Some topics, such as optimal diet for patients with IBD, lack rigorous scientific evidence [34]. In addition, much of the available health information appears to be difficult for patients to fully understand; for example, some information contains blood sample reference values with which readers may not be familiar, while the provided disease information may simply be too dense for a patient group [28]. *Health literacy* refers to an individual’s knowledge, skills and motivation to access, understand, apply and evaluate health information [35]. Human and environmental factors are important in anticipating how patients will understand and use health information, monitor their condition and interact with health services [36], making health literacy largely context dependent. *Digital health literacy* refers to patients’ ability to search for, find, understand and critically appraise information from digital resources to promote their own health [37]. Despite its association with health outcomes [38], health literacy among IBD patients has thus far been inadequately studied [39], and to our knowledge, no previous studies have examined the digital health literacy of IBD patients.

IBD patients have reported a lack of adequate explanations from HCPs about the diagnosis and treatment of IBD [40]. Concerns have been reported related to HCPs’ interest in collecting information about patients’ experiences of their symptoms [41], HCPs’ misinterpretation of reported symptoms [42] and the difficulties faced by patients in sharing emotional and intimate concerns with their HCPs [17]. However, both IBD patients [40] and HCPs [17] have acknowledged the need for an application that allows them to communicate with each other about the patients’ health status.

The effective provision of high-quality, relevant health information content to patients through digital media—for example, on topics such as medicinal products—may enhance and facilitate everyday self-management [43] and drive patients’ choice to adhere to treatment [20]. Many IBD patients use the internet as a source for health information [44], but much of this content is unregulated or unsupervised, and may be inaccurate, possibly misinforming and bewildering individuals looking for health information [2, 40]. Because of the importance of giving adequate attention to helping individuals facilitate their own self-management via digital services [45], systematic efforts are necessary to effectively communicate high-quality, relevant health information content to support IBD patients’ engagement in their own health and healthcare activities. In turn, to develop digital approaches to enhance the delivery of health services, there is a need to involve patients and providers [40, 46].

The current study is part of the public–private partnership Gravitate-Health, funded under the Innovative Health Initiative [47]. Gravitate-Health aims to establish an open access digital platform with tools and services that provide individual citizens better access to relevant health information from trusted sources; then, Gravitate-Health will investigate whether improved access to and understanding of treatment affects adherence, acts as a risk minimisation measure or improves treatment results and quality of life. This part of the Gravitate-Health initiative seeks to develop an open access digital platform and pilot the functionality of a digital health information service that offers reliable, personalised and tailored health information to meet IBD patients’ demands for relevant health information and health services that augment their self-management activities. Specifically, the aim of this study is to contribute to the development of such a platform by eliciting IBD patients’ health information needs, identifying the preferred and valued sources among IBD patients and their HCPs, and determining key aspects of the digital approach that promote self-management.

This study addresses the following research question: *How can digital health information be prioritised and focussed to support everyday self-management activities among patients with IBD?*

## Methods

Situated in the philosophical branch of critical realism [48], this mixed methods study was conducted within the paradigm of pragmatism [49]. Three different methods were used to collect data, with overlapping data collection. Data from a hospital’s internal audit for self-review and improvement were extracted, reviewed and analysed. The audit retrospectively examined electronic health record (EHR) summaries from interactions between patients and HCPs through a clinic’s IBD help line in the period from January 2020 to December 2022. Semi-structured interviews with IBD patients were also conducted, and two focus group interviews were conducted with HCPs. In this study, the term HCP primarily refers to gastroenterologists and nurses, but also other individuals with a healthcare profession, involved in the provision of IBD healthcare services.

### Sample and recruitment

The study took place in two outpatient clinics, which together serve more than 1,800 IBD patients, in a gastroenterology department at a large university hospital in Norway. Only IBD patients over the age of 18 were included in this study. The audit included all available and relevant EHR notes from the IBD help line at the outpatient clinic responsible for patients not receiving biological treatment.

A study nurse recruited and informed participants who took part in the interviews. Using purposive sampling for both patients and HCPs, participants were selected based on characteristics that could provide heterogeneity and insights to the study [50, pp. 75–80, 51, p. 254]. For patients, this involved variations in age, IBD diagnosis, disease severity and medical treatment. Among HCPs, this meant including gastroenterologists and nurses with varying responsibilities within the department. HCPs were only included if they had a license and were involved in the treatment, care and follow-up of patients with IBD. Interview participants without the ability to give confirmed consent or to express themselves in Norwegian, Danish, Swedish or English were excluded.

### Data collection

#### Audit extraction

With the purpose of controlling and improving an activity, an *audit* is defined as an investigation into whether an activity meets explicit standards [52, p. 14]. The IBD help line is a service that provides information and advice on IBD treatment and related issues for patients of the associated hospital who do not receive biological treatment or frequent follow-up. The notes from interactions via the help line are registered in an EHR. The audit being analysed in the present study was conducted from October 2022 to January 2023.

A protocol and an online codebook were initially developed in close collaboration with a nurse. The codebook was first tested with 25 notes to assess acceptability for data collection; it was then revised and finalised with specific categories.

#### HCP focus group interviews

The focus group interviews were conducted in Norwegian in the period from October 2022 to February 2023. Aiming to ensure effective data collection [50, p. 152], the HCPs were divided into two separate focus groups, and the interviews were conducted face to face at the hospital.

#### Patient interviews

Patients were interviewed individually, during the same period. Two separate interviews guides were developed—one for IBD patients and one for IBD HCPs (see Appendix 1 and 2). Those receiving biological treatment were interviewed at the hospital before, during or after treatment. Patients who did not receive biological treatment were interviewed either face to face, via a telephone or video call, depending on their preference. All interviews were audio recorded with a Zoom H4n Pro Audio Recorder and transcribed for thematic analysis (TA).

### Data analysis

#### Statistical analysis

Of the the audited EHR, 10% were independently assessed by 2 coders for interrater reliability and were deemed appropriate, with at least 80% agreement and Cohen’s kappa values above 0.60 [53]. Using SPSS Statistics version 29, coded data from the audit were examined through frequency distributions, and for each independent variable, differences between groups were analysed using Pearson’s chi-squared test.

#### Qualitative thematic analysis

**Fig. 1:**
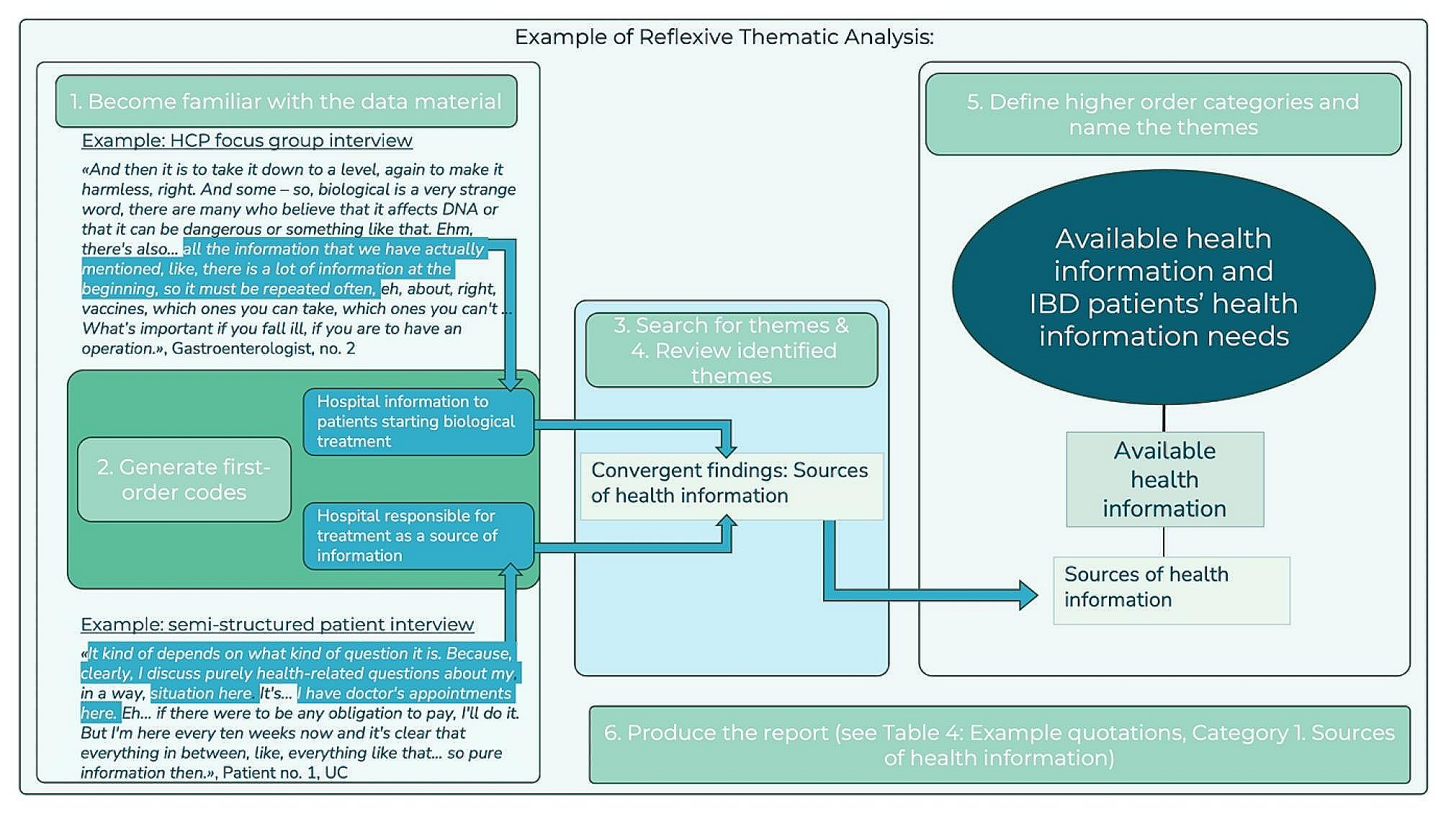
Example of reflexive thematic analysis following the guidance of Braun & Clarke [54, 55]

Using the NVivo software, transcribed qualitative data were analysed through reflexive thematic analysis (TA). Although emphasised as more of a recursive process than a linear one, TA consists of six phases, which we followed: (1) become familiar with the data material, (2) generate first-order codes, (3) search for themes, (4) review the identified themes and (5) define higher order categories and name the themes before (6) producing the report [54, 55]. The data material were initially triangulated by sorting data from the focus group interviews and the semi-structured patient interviews into two separate files, respectively. From step three onwards in the TA, the relationship between findings from the two methods were considered [56, 57]. Figure 1 provides an example of the progression of in the thematic analysis in this study. As the interviews were conducted in Norwegian, the quotes were translated into English for the presentation of the results.

## Results

### Audit

For this study, the audit data material consists of 1,481 journal notes from the IBD help line. As patients, in some cases, called in for several reasons during a single call, each documented reason to call was independently assessed. Table 1 provides an overview of the patient characteristics.

**Table 1:** Patient characteristics

Characteristics	*N* (%) of registered journal notes 1481 (100)
Age	
18–25 26–45 46–65 > 65	111 (7.5) 696 (47.0) 508 (34.3) 166 (11.2)
Gender	
Male Female	536 (36.2) 945 (63.8)
Diagnosis	
Crohn’s disease Ulcerative colitis	387 (26.1) 1094 (73.9)
Duration of disease	
1 year or shorter 1–5 years > 5 years	84 (5.7) 693 (46.8) 704 (47.5)

Female patients constituted more than 60% of the total sample, representing the majority of patients for both diagnoses (UC = 77.5%; CD = 59.0%). Nearly three-quarters of the registered patients in these journal notes had UC. Another noticeable finding was that patients diagnosed within the last 5 years, made up nearly 50% of the total registered journal notes. Over half of the sample was 45 years old or younger at the time the calls took place.

The documented reasons for contacting the hospital are presented in Table 2.

**Table 2:** Reasons for contacting the hospital via the IBD help line

	1. Symptoms (%)	2. Medicines (%)	3. Examinations and tests^a^(%)	4. Other reasons^b^(%)
**Total**	523 (100)	263 (100)	796 (100)	41 (100)
**Gender**	*p <* .01	*p <* .05	*p <* .001	*p* = .70
Male	213 (40.7)	111 (42.2)	250 (31.4)	16 (39.0)
Female	310 (59.3)	152 (57.8)	546 (68.6)	25 (61.0)
**Diagnosis**	*p <* .01	*p <* .001	*p <* .001	*p* = .06
Crohn’s disease	114 (21.8)	44 (16.7)	248 (31.2)	16 (39.0)
Ulcerative colitis	409 (78.2)	219 (83.3)	548 (68.8)	25 (61.0)
**Age**	*p <* .01	*p <* .05	*p <* .001	*p* = .59
18–25	53 (10.1)	30 (11.4)	42 (5.3)	1 (2.4)
26–45	255 (48.8)	120 (45.6)	363 (45.6)	22 (53.7)
46–65	164 (31.4)	92 (35)	289 (36.3)	14 (34.1)
> 65	51 (9.8)	21 (8)	102 (12.8)	4 (9.8)
**Disease duration**	*p* = .59	*p <* .001	*p <* .001	*p* = .41
< 1 year	34 (6.5)	27 (10.3)	29 (3.6)	3 (7.3)
1–5 years	242 (46.3)	131 (49.8)	373 (46.9)	15 (36.6)
> 5 years	247 (47.2)	105 (39.9)	394 (49.5)	23 (56.1)

^a^ Includes requests for, information about and answers to different examinations and tests; ^b^ includes coronavirus (COVID-19; *N* = 14), diet (*N* = 10), information to general practitioner (*N* = 3), stoma management (*N* = 2), general IBD information (*N* = 6), social services (*N* = 2), study participation (*N* = 1), diagnosis (*N* = 1), kidney stones (*N* = 1) and unspecified (*N* = 1); Cohen’s kappa (κ) and percentage of agreement for registered reasons: symptoms (κ = 0.81, 91.9%), medicines (κ = 0.67, 87.2%), examinations and tests (κ = 0.70, 85.1%) and other reasons (κ = 0.47, 90.5%).

The most frequently documented reason for contacting the hospital was to ask for examinations and tests—such as faecal tests, endoscopic examinations and blood samples—followed by reporting symptoms, including those that had recently occurred and those that were persistent. Less than 20% of all registered calls reported in the journal notes took place with the purpose of talking about medicines, and slightly less than 3% of registered calls were initiated for reasons other than symptoms, medicines or scheduling examinations and tests.

When analysing the distribution of patient characteristics, differing patterns emerged. Among male patients, there was a larger internal proportion of calls related to symptoms and medicines but a smaller internal proportion of calls for examinations and tests, compared to female patients. When comparing CD and UC patients, a larger share of the CD patients called in to the hospital for examinations and tests, while a larger share of the UC patients called in for symptoms and medicines. When examining the internal proportion of calls in relation to age group, there appeared to be a sinking trend, with less patients calling in for symptom-related inquires as age increased. Although not as clear, this tendency was also identified for medicine-related calls. The tendency was the opposite regarding age and calling in for examinations and tests, as a larger proportion called for this reason within each increasing age group.

A higher proportion of recently diagnosed patients called about symptoms compared to patients diagnosed more than five years ago and to patients diagnosed within the last one to five years. The same tendency was present when the reason for the call was medicines. In terms of patients’ disease duration and whether they called for examinations and tests, a larger proportion of patients diagnosed more than five years ago called for examinations and tests compared to patients diagnosed within the last one to five years and recently diagnosed patients.

### Interviews with patients and HCPs

In total, 17 patients were recruited to participate in semi-structured interviews, while a total of 11 HCPs were recruited for 2 focus group interviews. Table 3 provides an overview of the characteristics of the participants interviewed.

**Table 3:** Sample characteristics, participant interviews

**Patients**	
**Age**	Median age: 47 Range: 22–70 years old
Gender	7 male 10 female
Diagnosis	9 ulcerative colitis 8 Crohn’s disease
Medical treatment	10 receiving biological treatment^a^ 6 receiving 5-aminosalicylic acid treatment 1 receiving no treatment
**Healthcare Professionals**	
Focus group 1	3 gastroenterologists 3 nurses
Focus group 2	2 gastroentereologists 3 nurses

^a^The biological treatments include infliximab, adalimumab, vedolizumab and ustekinumab

**Fig. 2:**
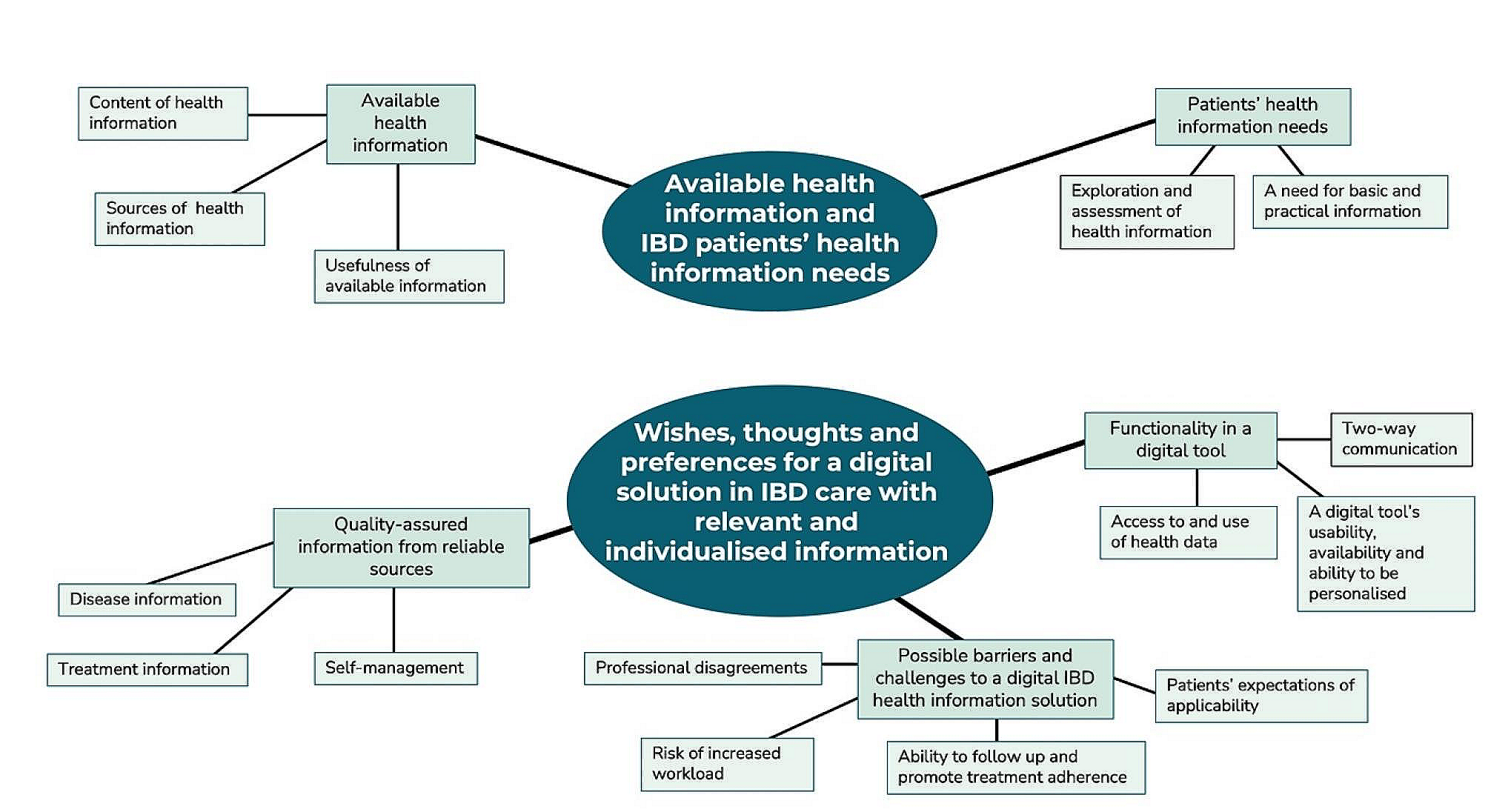
Identified themes with associated sub-themes and categories from the qualitative analysis of interviewed participants

Two major themes were identified from the qualitative analysis: (1) the available health information and IBD patients’ health information needs, and (2) whishes, thoughts and preferences for a digital solution in IBD care with relevant and individualised information. Figure 2 provides an overview of the identified themes.

#### The available health information and IBD patients’ health information needs

Table 4 provides an overview of the identified categories under the theme of the available health information and IBD patients’ health information needs.

**Table 4:** Available health information and IBD patients’ health information needs

	**Categories**	**Example quotations**
**Available health information**		
	**1. Sources of health information** Mentioned general sources for information were HCPs, public online resources and social media.	*‘It kind of depends on what kind of question it is. Because … I discuss purely health-related questions about my … situation here. … I have doctor’s appointments here’.* (Patient 1, UC)
		*‘All the information that we have actually mentioned, like, there is a lot of information at the beginning, so it must be repeated often’.* (Gastroentereologist 2)
		*‘No, I’ve tried some apps like some … international … I don’t remember what they’re called, but … more like IBS-type stuff’.* (Patient 10, CD)
		*‘I have a good friend with ulcerative colitis. … I used her very much as a kind of encyclopaedia for ulcerative colitis when I was diagnosed, because she has had it for almost ten years and has been through a lot’.* (Patient 16, UC)
		*‘Some are active on Facebook groups … willing to get involved in, “I have this experience that has worked for me”, which is very little like … quality assured … and it is … emotionally engaging, stories like that, so you understand that you are going there, but …’* (Gastroentereologist 1)
	**2. Content of health information** Patients sought information based on needs. HCPs tailored medical information.	*‘So, the main info I got in my kind of acute phase … it was actually in relation to coping and … how to live with it’.* (Patient 4, CD)
		*‘We don’t necessarily agree with what’s written … because those package leaflets are made for legal purposes. … These can be very difficult decisions, and not necessarily something you can read up on in a package leaflet, right?’* (Gastroentereologist 2)
	**3. Usefulness of available information** Limitations of available health information were pointed out.	*‘One more thing, which very many patients struggle with and which we have very limited information about is fatigue’.* (Gastroentereologist 2)
		*‘What dietary advice can be given for IBD? That’s what makes it so difficult—that there isn’t any’.* (RN 1)
		*‘Often, it turns out that you have to read like two pages before you get some … insight, but you haven’t got a complete answer’.* (Patient 14, UC)
		*‘We have such a high level of education and sometimes it is so difficult to meet the ability to receive information, that such general information to the population … should probably be at the secondary school level’.* (Gastroentereologist 4)
		*‘How long it is usual to have an active inflammation before it begins to calm down? I have not found that anywhere. Or [I find] … “It can last from a few days to several years”, so it’s like, “Great comfort …”* (Patient 17, UC)
**IBD patients’ health information needs**	**4. A need for basic and practical information** was identified in terms of disease, treatment, precautions in relation to medicines, diet, social rights and other topics related to self-management.	*‘I really wondered about everything. … Can you have a family with the disease? Is it hereditary? How to avoid stress, what to eat …’* (Patient 2, CD)
		*‘Is this something that will somehow get worse over the years, is it something that I should be careful about over the years? After all, I want to have a good life’.* (Patient 14, UC)
		*‘What is biological treatment? … I don’t know, and no one has said anything about it either’.* (Patient 6, CD)
		*‘It has been such a long time since people have travelled’.* (RN 2)
		*‘My claim is that there are as many paths in diet as there are people who are exposed to the disease … so I think you could gain a lot from … trying to talk a little about it in public’.* (Patient 15, UC)
		*‘What rights and offers … exist … it could have been more organised … both in relation to rights against NAV and … diet and nutritionists and things like that’.* (Patient 8, CD)
		*‘How much to take, what type of tablet to use … you just get to know that, yes, you’ll just have to find out for yourself’.* (Patient 5, UC)
		*‘Something like, “Usually, we tend to do it like that’. … I guess I miss that … instead of having to call … and then I am told that, ‘Yes, you were actually supposed to come in October”’.* (Patient 11, CD)
		*‘Sometimes you might wonder a little about tests … you’re actually waiting to get an answer, but you don’t get it’.* (Patient 8, CD)
		*‘How many end up with a stoma? … How bad do you have to be before … it’s a topic? … How often can you … expect to get inflammation?’* (Patient 17, UC)
	**5. The exploration and assessment of health information** appeared related to patients’ disease duration and activity. HCPs related information needs to treatment effect.	*‘You look for information the most when you are either new to it or … the disease is active, that you are very bothered by it’.* (Patient 3, CD)
		*‘If they [patients] have a good effect [of treatment] and are reasonably asymptomatic, then they are not that interested … but if they … experience that they are not getting the help they would ideally like, then they become very open to all possible alternative and undocumented sources’.* (Gastroentereologist 3)

*Abbreviations: IBD*, inflammatory bowel disease; *IBS*, irritable bowel syndrome; *UC*, Ulcerative colitis; *CD*, Crohn’s disease; *NAV*, Norwegian Labour and Welfare Administration; *RN*, registered nurse.

##### Available health information

***Sources of health information.*** The patients were familiar with digital solutions focussed on overall health or other medical conditions. Many referred to digital resources when looking for information, mostly via the internet. Patients sought health information largely from the healthcare system, which was aligned with HCPs, who also identified themselves as the patients’ main provider of health information, although this information appeared to be primarily communicated verbally. Regarding digital health services, only one patient was identified as using an IBD-specific solution. Patients also talked about sharing information and their own experiences with others via social media and with colleagues, family and friends. However, social media was also pointed out by HCPs as a source of potentially misleading information.

***Content of health information.*** The health information patients searched for was based on their situational needs. The content largely dealt with IBD treatment, what IBD is and how to manage the disease pragmatically. In some cases, gastroenterologists provided tailored medical information as an alternative to the package leaflets that come with prescribed medicine, as these were rarely perceived as easily understandable for IBD patients.

***Usefulness of available information.*** HCPs and patients both pointed out the limitations of the available health information, both in terms of its availability and quality, on topics such as fatigue, diet, symptoms and disease activity.

##### IBD patients’ health information needs

The interviewed patients expressed a need for information in the context of their disease, ranging from information surrounding their disease and its treatment to that related to self-management.

***Need for basic and practical information.*** In general, patients reported a need for more basic information about the disease—including its aetiology, progression, duration and symptoms—as well as information related to self-management, such as social security and how to maintain a satisfactory quality of life. Patients specifically wanted more accessible information about what to expect from the disease in terms of symptoms, progression and what other people have experienced. Both the HCPs and patients expressed a need for the provision of practical patient information focussing on how to manage the disease both in clinical care and in daily living, including topics such as nutrition, travel and symptom management. Patients further called for information about treatment, particularly in terms of their own responsibilities, medical treatment, test results and treatment progress.

***Exploration and assessment of health information.*** Many patients reported a need for information at the time of diagnosis. While patients identified a relationship between their exploration of IBD information and how recently they were diagnosed or how badly they were affected, HCPs acknowledged that patients’ need for information tends to depend on the outcomes of their treatment.

#### Wishes, thoughts and preferences for a digital solution in IBD care with relevant and individualised information

Table 5 provides an overview of the identified categories under the theme of whishes, thoughts and preferences for a digital solution in IBD care with relevant and individualised information.

**Table 5:** Wishes, thoughts and preferences for a digital solution in IBD care with relevant and individualised information

Quality-assured information from reliable sources	
Categories	Example quotations
**1. Disease information** General information about the disease, possible outcomes and symptoms, and their frequency	*‘If you collected all … frequently asked questions by IBD patients, then you could perhaps get far by just doing that’.* (Gastroentereologist 3)
	*‘They need to know what is wrong with them and what the goal of the treatment is’.* (Gastroentereologist 4)
	*‘[It is important for patients to know] that you can have … residual symptoms, persistent chronic symptoms despite successful treatment and being able to understand that … there is not always a short-term medical solution to this’.* (Gastroentereologist 2)
**2. Treatment information** Information related to relevant examinations and tests, and the use of medicines, such as duration, precautions, changes, administration, antibiotics and importance	*‘The faecal test … they should learn … what it means, what consequence it has. … I think it would have been a great advantage if that was available’.* (RN 1)
	*‘What is it [the medicine] … what does it do technically, side effects are important. … It could be interesting to have a simple overview’.* (Patient 1, UC)
	*‘Injection technique … the difference between pen and syringe … pay attention to hand hygiene …’* (RN 2)
	*‘Some information about the use of antibiotics … there are a lot of patients who are very anxious about starting antibiotics for various reasons’.* (Gastroentereologist 1)
	*‘The importance of not making changes on your own. … There could be side effects, but there doesn’t have to be. … Contact your doctor if you think you’re going to make changes’.* (Gastroentereologist 4)
**3. Self-management** Recommendations for managing the disease in patients’ daily lives, and information about existing health and social services	*‘Just that overriding sentence that “You don’t eat yourself healthy”. … You don’t heal yourself with diet and you don’t eat yourself sick … but it will be [helpful] to alleviate the ailments you have’.* (RN 1)
	*‘What do you do if you travel? … What do you do if you get sick? … How many doses can you take of cortisone?’* (Patient 13, UC)
	*‘I think it’s a bit far-fetched that we can say that they can get early offers from mental health care’*. (Gastroentereologist 3)
	*‘I feel that many of them need help to… shift the focus. … What are your resources, what is your network, what support do you have?’* (RN 2)
	*‘It would have been nice for me to meet someone the same age who has the same disease. … There must be more of us’.* (Patient 17, UC)
**Functionality in a digital tool**	
***Categories***	***Example quotations***
**4. Two-way communication** Allows HCPs and patients to communicate and make appointments over a digital platform	*‘They know everything about me, they have the patient records and they know what has happened. So, if I were to have a general question about fatigue or fistulas … it would be good to ask’.* (Patient 9, CD)
	*‘You need to have a filter.… If you are constantly available then you will be disturbed all the time and that doesn’t work either’*. (Gastroentereologist 3)
	*‘Easily accessible appointments … because we have a lot of people who we have to get here, because they don’t remember when they have an appointment’*. (RN 2)
**5. A digital tool’s usability, availability and ability to be personalised** The ways in which a digital tool can be personally adapted with satisfactory usability (i.e., language, security and simplicity)	*‘It might be a point to perhaps get the completely general information in several languages as well’.* (RN 3)
	*‘The first time you log in with it [your unique portal credentials], you also have a personal code of four digits’*. (RN 2)
	*‘It must be something that you can pull up in slightly different contexts’.* (Patient 15, UC)
	*‘If it’s an app … and you get a reminder … to take your medicine … then it will help with compliance, I think’*. (Gastroentereologist 1)
	*‘I’m always very keen to reduce the information. [Asking, ] “What is important in this text?” and then removing everything that is a filler’.* (Patient 5, UC)
	*‘There is a [interaction] notification in [the EHR] when you try to print … but if it’s not in the hospital, you don’t get that alarm’.* (Gastroentereologist 5)
**6. Access to and use of health data** The ability to track received treatment, symptoms and disease activity markers	*‘Some kind of timeline or something … you can … forget a bit … it would be nice to have’.* (Patient 10, CD)
	*‘There is a reason why test answers are not published. … If we first publish it, we have to follow up on all this as well’*. (RN 1)
	*‘I don’t bother to sit and read my medical record or what some doctor refers to what I have said or how I act … but just things that … would be directly measurable for me based on how I feel, just so that I could sort of tune my own situation. … That’s the main wish’.* (Patient 11, CD)
	*‘I think many people want to have [the faecal test results] available’*. (RN 2)
	*‘What I would be most interested in as a therapist is something like simple symptom scores to follow the patients not only when they are asked directly here’*. (Gastroentereologist 2)
	*‘It’s also something … I think could have been very valuable … to have somewhere to log when things are going well, when things are going bad and which factors possibly influence the disease’.* (Patient 16, UC)
	*‘Such as, for example, the faecal tests—I wish I could have seen the graphs myself … because then I would have had a relationship with it in a way. … I often have to ask specifically, “Yes, but what was the value?”’* (Patient 7, UC)
**Possible barriers and challenges to a digital IBD health information solution**	
***Categories***	***Example quotations***
**7. Professional disagreements**	*‘It’s not like every doctor in the whole country agrees with how we manage things here’*. (Gastroentereologist 1)
**8. Risk of increased workload**	*‘The challenge with websites is that they must be maintained. … It must be an administration’*. (Gastroentereologist 4)
	*‘I think that for busy clinicians, having yet another place to log in and yet another portal or something to learn—it can mean that you don’t necessarily want to use it*’. (Gastroentereologist 1)
**9. Ability to follow up and promote treatment adherence**	*‘When they don’t feel bad, they give less compliance … and they forget the whole disease’*. (Gastroentereologist 3)
	*‘When we talk about adapting information, it is based on how we perceive the patient to be as a personality, or [based on] an … active part of the disease. And these are things that will be difficult for a platform to understand’.* (Gastroentereologist 2)
	*‘Younger men—or at least the IBD patients—they are the ones who are not doing well—the men who pretend they are not sick’*. (Gastroentereologist 4)
**10. Patients’ expectations of applicability**	*‘I think that a digital service where you can gather all the information and have some form of messaging service could have been very useful’.* (Patient 16, UC)
	*‘When it comes down to it, I’m not that interested anyway, but it i*s *… a thing that perhaps should have existed’.* (Patient 8, CD)

*Abbreviations*: *EHR*, electronic health record; *IBD*, inflammatory bowel disease; *UC*, ulcerative colitis; *CD*, Crohn’s disease; *RN*, registered nurse.

##### Quality-assured information from reliable sources

Both patients and HCPs reflected on possible useful services and functions that could be included in a digital solution. The HCPs emphasised the importance of creating a tool that is easy to use and that is well integrated with the treatment that patients are already receiving. The HCPs also expressed the desire to be able to select core information about the disease and available treatments to direct patients to, with different levels of immersion available under each subject.

***Disease information.*** Both the HCPs and patients mentioned the benefit of having a simple overview of recurring questions easily available. Another point made by patients was that the information provided should be up to date. One gastroenterologist stressed the importance of including information about fatigue, as the information offered on this topic was perceived as insufficient. Another gastroenterologist saw such a platform as an opportunity to communicate the potential limitations of the medical treatment of IBD symptoms.

***Treatment information.*** In terms of treatment, HCPs emphasised the importance of including different informational aspects related to medicine to consider, such as precautions, changes in medicine, administration, antibiotics and the importance of taking prescribed medicine. Patients also called for information about medicine, both in terms of practical and relevant information and in terms of possible alternatives.

***Self-management.*** Patients expressed a preference for a tool that provides quality-assured information about living with IBD from reliable, up-to-date sources, containing overviews on different services and disease information. They also wanted access to more information about the available health and social services. However, in terms of the available services, the HCPs pointed out the potential limitations and, in contrast to the patients, called for information that promoted greater initiative on the patients’ part in the form of self-management. Patients also called for the opportunity to contact peers to share experiences and learn from each other. The HCPs agreed that this would be very beneficial to patients.

##### Functionality in a digital tool

***Two-way communication.*** Two-way communication—that is, a service providing both patients and HCPs the opportunity to contact each other—was a topic on which both HCPs and patients shared reflections. Some believed a communication channel would benefit the healthcare service and the self-management of patients, while others saw it as an opportunity to get in touch more easily. However, some also pointed out the importance of not increasing the workload of HCPs through such a tool. Despite this reservation, through digital communication, both HCPs and patients pointed out the benefit of making appointments in the tool.

***A digital tool’s usability, availability and ability to be personalised.*** Patients and HCPs reflected on how a digital health tool could be adapted to each patient’s unique situation with satisfactory usability. The HCPs pointed out the necessity of patients having their own user profiles, with a secure but simple design. Patients, in contrast, were concerned with how time consuming and relevant such a service would turn out to be for them. Both HCPs and patients wanted the tool to be available as a mobile phone application with the ability to send reminders, especially to help promote adherence to treatment plans and medication schedules. Considering the potential list of medications that could be prescribed to patients, which was envisioned to be included in the platform, one MD pointed out the importance of having a function for the provision of interaction notices.

***Access to and use of health data.*** All patients had some reflections about their access to their own health data. Many patients expressed the great benefit of having the ability to track received treatment, symptoms and disease activity markers, such as faecal tests. The HCPs expressed the benefit of allowing symptom tracking for patients but also pointed out the challenges inherent in making some data available. For example, one registered nurse (RN) expressed the concern that the increased availability of blood sample test results would imply an expectation from patients that HCPs will address these test results to a greater extent than they are initially able.

##### Possible barriers and challenges to a digital IBD health information solution

Several challenges were also identified when it came to disseminating information to patients through a digital tool. HCPs, in particular, expressed concerns about a lack of clarity for patients and increased workload for HCPs, as well as the ability to provide individually tailored, sound information when some elements of this information may still be professionally disputed. Some also expressed concerns that digitalisation might change or reduce the ability of HCPs to follow up with patients and promote treatment adherence. Young men, for example, were identified as a group of patients who showed little willingness to follow up with treatment, and this may be exacerbated if they rely too heavily on a digital platform for information. The patients expressed differing views on having a digital tool to promote their self-management—some welcomed it with great enthusiasm, while others were more sceptical.

## Discussion

To our knowledge, this is the first study to make use of real-world data from an EHR audit combined with HCP and patient interviews to explore health information needs and guide how to prioritise information in digital health information services to promote the self-management of patients with IBD. As the following sections illustrates, the study demonstrated interesting findings that complement those of previous studies.

### Prioritisation of digital health information services to promote the self-management of patients with IBD

Consistent with previous findings [2, 40], both the patients and HCPs participating in this study identified the healthcare service as the main source for patient health information. Most notes dealt with examinations and tests, with patients requesting test results, seeking information about the various tests available or scheduling appointments for examinations. It appeared to be important to patients that information about these topics be prioritised in a digital solution in order to facilitate optimal self-management. Combined with the wish communicated by all interviewed patients for the ability to track their disease activity markers, their own treatment and their own symptoms, these findings correspond to the previously identified need for patients to have access to more information to facilitate self-management in their daily lives [28, 31]. Most remaining calls considered symptoms and medicines, complementing the previously reported limitations in the ability of IBD patients to access information about these topics [32, 33].

The interviewed patients also expressed a need for basic and practical IBD health information that is personalised, tailored and aimed at promoting self-management. However, the HCPs argued that they did already adapt the medical information they provided to the patients’ individual needs. This incongruity may be a result of limited awareness among patients of the health service’s information offering. HCPs may also misinterpret reported symptoms [42] and, thus, provide inadequate information on diagnosis and treatment [40]. In addition, the HCPs appeared to mainly hand over health information verbally, not in writing or digitally, possibly limiting the patients’ access to what they consider their most reliable source of health information. Patients’ ability to understand health information was a recurring challenge expressed by the HCPs in the focus group interviews, as they indicated that patient information often had to be repeated. Another important aspect pointed out by the HCPs was that patients may perceive health information as inadequate if the effect of their treatment is insufficient. Still, both the patients and HCP expressed a need for available situation-specific patient information about the disease, treatment and self-management. Together with the registered reasons for contacting the hospital, these findings argue for the prioritisation of relevant patient information about medicines and symptoms.

Few notes in the sample discussed diet and nutrition, despite the previously reported need for information related to this topic [28, 32]. The interviewed patients did, however, discuss the need for information about diet and nutrition, although some were convinced that practical information on this topic was limited. The HCPs stressed the importance of patient awareness about the difference between the treatment characteristics of nutrition and the properties of nutrition as a symptom-relieving measure [34]. One explanation for this conflicting finding may be that patients in general call the hospital with questions regarding their medical treatment, as this is the hospital’s area of responsibility. However, patients expressed in the interviews that they seek out information about self-management activities, such as diet, on their own. If that information is not collected from the healthcare service, they tend to refer to the internet; this was a finding from both the interviews and the literature [44]. This may give cause for concern, considering the lack of available diet information expressed by patients [28, 32] and considering the findings in the literature confirming HCPs’ perceptions of the internet’s limited ability to supress unregulated and unreliable sources [2, 40].

### Focussing digital health information services to meet individual IBD patients’ needs

In general, patients wanted a digital tool that provides them with information about health and social services that are relevant, up to date, reliable and practical for their self-management of IBD. As reported in previous studies, IBD patients request information that meets their needs, either by helping to prevent relapse [27, 28, 32, 33] or to aid them on their way back to remission [30–32]. The perception of the healthcare service as the main source for disease-related health information implies a responsibility from the healthcare service as a provider of this information, even if that information is digital in nature. As such, the HCPs expressed concern about how to provide high-quality health information that is understandable, relevant and adapted to patients. The currently available health information is not necessarily adapted to patients’ health literacy [28], and information from HCPs may need to be mediated to ensure this [40].

Although called for by patients [28], some information was deemed by HCPs as being difficult to provide to patients, such as results from blood samples, as this may add a considerable workload to account for all possible implications. However, making faecal test results and a simple symptom score accessible to both patients and HCPs were embraced by all interviewed patients and HCPs as practical for patient self-management and IBD treatment and follow-up. Ideas for how relevant information could be focussed and prioritised were also posited, such as the suggestion to have different levels of immersion for each subject, depending on each patient’s needs. Another suggestion was to focus the patient health information on the categories of disease and treatment. No suggestions were identified to how health information of relevance could be targeted towards individual patients.

Although the results were somewhat ambiguous, the chi-squared analysis of the registered reasons for contacting the hospital may imply different needs for focussing information, depending on different characteristics. This study found that CD patients, compared to UC patients, requested more information about examinations and tests, and less about symptoms and medicines; however, previous findings indicated that UC patients appeared more eager for practical information for daily living, while CD patients seemed more concerned with the risks associated with their disease [27]. To account for this difference, it is important to point out the skewed distribution of UC and CD patients in the current sample. In general, a higher proportion of patients with CD receive biological treatment compared to UC patients [58], and this was also the case for the current study’s sample population. The IBD help line is only available to patients not receiving biological treatment.

The larger proportion of female patients compared to male patients calling for information about or requests for different examinations and tests may confirm their previously identified orientation towards seeking out information concerning daily self-management [27]. However, it would then be reasonable to expect that a larger proportion of female patients compared to male would call the hospital about symptoms and medicines; this is contrary to our findings, as male patients proportionally called in more frequently than did female patients about these topics. Nevertheless, disproportionally more females than males called the hospital during the registered period, despite an evenly distributed prevalence of IBD between Norwegian male and female patients [59].

A similar issue can be identified in the relationship between age and calling the hospital about symptoms, medicines and examinations and tests. A larger proportion of younger patients appeared to call more frequently about symptoms and medicines, possibly confirming their previously identified orientation towards seeking out information concerning self-management [27]. Still, a larger proportion of older patients appeared more eager to call for examinations and tests. It is also possible that there is a connection between age and disease duration. While patients with a longer disease duration appeared to have a greater need for information about examinations and tests, recently diagnosed patients appeared to seek more information about medicines. This may imply that more experienced patients have a greater understanding of what the disease entails, reducing the need for general information. The patient interviews confirmed previous findings [28–30], as the patients expressed that the extent of their need for health information is greater at the time of diagnosis and during active disease; this is aligned with the aim of the IBD help line. Although the results from the IBD help line may provide equivocal findings, the overall findings appear to underline patients’ informational needs about topics that are beneficial for situational self-management, especially if they were recently diagnosed [28, 29] or were experiencing periods with greater disease activity [30].

### Considerations for the design and implementation of a digital health information platform in IBD treatment

Good communication strategies may improve the sharing of health information [5, 17], but the HCPs did not unconditionally deem a digital information service and communication channel as suitable for this aim. Rather than a substitute, they appeared to experience digital health information as an addition to the information they provide in consultations. For patients, a digital platform with relevant and reliable health information may facilitate treatment adherence [20] and self-management [43], and the HCPs pointed out possible functions within such a platform that may promote this. However, the HCPs also expressed concerns about their workload should they also have to manage an interactive digital solution within which information is updated when needed and through which patients have opportunity to contact their HCPs when needed.

It was also pointed out that the question of whether a digital health information platform would increase the workload of HCPs may depend on how well it is incorporated into existing digital services, such as the EHR. What is useful to end users in a system depends on their needs and preferences [26]; here, in addition to HCPs, the patients themselves are the end users, and they must rely on their own self-management to control their disease [12, 13]. In line with previous findings [21–24], patients deemed a digital platform as being beneficial for their self-management. The concern about the reduced ability of HCPs to promote treatment adherence among patients should justifiably be considered in the development of a digital health information platform [8]. However, if a digital tool provides patients with disease information and facilitates the monitoring and managing of disease-related challenges, it is not inconceivable that this, too, will promote self-management [43] and treatment adherence [20]. Subsequently, this may reduce the burden on the healthcare service [20], giving HCPs the opportunity to prioritise other tasks, such as providing and maintaining health information to patients.

Like the HCPs, the interviewed patients did not want a service that demanded more work from them, as they articulated a need for services that facilitate their everyday life—not those that complicate it. Although some IBD patients also expressed concerns about a digital open communication channel between patients and HCPs, most appeared positive towards the idea. Further, none of the interviewed patients thought that they would burden the healthcare system with their health information needs through such a communication tool; the patients seemed to view this as an opportunity for more balanced communication, as they can turn to the application for information when they are feeling worried rather than possibly making unnecessary contact with the hospital. As such, a digital platform could ease their concerns about becoming a burden to the healthcare service. The patients themselves identified this as a way to overcome previously reported challenges such as sharing concerns with HCPs [17] and receiving inadequate explanations [40].

The combined findings of this study provide guiding recommendations for developing a digital tool offering personalised and tailored IBD health information to augment patients’ self-management activities. Based on the results, Table 6 proposes recommendations to allow HCPs to prioritise digital health information content to support the everyday self-management of patients with IBD, as well as considerations for focussing on information that is tailored to individual patients based on their characteristics.

**Table 6:** Content to prioritise and focus on in a personal digital health information platform for IBD patients

Health information				
Frequently asked questions	Diagnosis*			
	*Disease*		Introduction	
			Progression	
			Prognosis	
			Complications	
	*Symptoms*		Very common	
			Common	
			Rare	
			Very rare	
	Treatment*			
	*Diagnostic tests* ^*a*^	Blood samples		
		Faecal tests		
		Endoscopic examinations		
		Other diagnostic tests		
	*Medical treatment* ^*b*^ Importance, duration of treatment, clinical information of relevance to individual patients’ medicine list	5-ASA Corticosteroids Immunosuppressants Biopharmaceuticals Antibiotics	Indication	Side effects
			Dosage	Overdose & poisoning
			Administration & instruction	Properties
			Contraindications	Storage & durability
			Precautions	Other information
			Interactions	
	*Progress plan*	Expected course of the individual’s treatment		
	Self-management*			
	*Practical recommendations for daily living*	Symptom management	When to seek health care	
			Fatigue	
		Symptom prevention	Diet	
			Smoking	
			Physical activity	
			Stress	
			Coping	
		Traveling	Preventive measures	
			Symptom management	
			Available healthcare services	
	*Resources*		Peer associations	
			Welfare services	
			Nutritionist	
			Psychologist	
			Social services	
Patient access and use of personal health data incorporated with the EHR*				
	**Personal health record with easily interpretable and systematically organised information**			
	*Treatment* ^*b*^	Medical treatment history		
		Surgical treatment history		
		Medicine list with reminder function, promoting treatment adherence		
	*Disease activity markers* ^*a*^	Faecal tests		
		Endoscopic examinations		
		Blood samples		
	*Possibility to supplement with the patient’s subjectively experienced symptoms*	Simple symptom score		
		Fatigue		
		Health-related quality of life		
Two-way communication incorporated with the EHR*				
	**Regulated messaging service with the hospital’s gastroenterology department**			
	**Individual patient schedule**			

*Considered desirable for all patient groups; ^a^ preferably highlighted for patients with a disease duration of more than one year; ^b^ preferably highlighted for patients with a disease duration of less than one year.

*Abbreviations: 5-ASA*, 5-aminosalicylic acid; *EHR*, electronic health record.

### Limitations

Mixed methods is an approach it can be difficult to assess due to limited consensus on quality standards. Three issues are consistently highlitghted in the literature. One issue is whether the use and integration of different methods is supported by the purpose of the study [60]. The authors believe the insights provided from this study could not been obtained using only one of the methods. Combining findings from both qualitative and quantitative data analysis [56], mixed methods is an approach that holds promise for addressing the complexity and context that follows innovation research [61]. The other two issues regarding the use of mixed methods are the quality of the individual results obtained from the different methods and the quality of the integrated findings [60].

Instead of quantifying the phenomena under study, qualitative methods seek to gain a more in-depth understanding of the phenomena and are therefore unsuitable for obtaining generalisable findings [50]. However, qualitative research acknowledges the experience of individuals in the context in question and can thus be very useful in the investigation of human interactions, experiences, thoughts and values [62]. Three specific issues have been reported to affect qualitative research: (1) transferability, the findings’ applicability outside the study circumstances, (2) reflexivity, awareness of own experiences, perspectives and position, and (3) the interpretation and analysis of data [63]. In terms of transferability, the relevance of this study will depend on both the authors’ and the individual reader’s perspectives and context [64, p. 192]. The integration of digital technology into health is often a complex, multifaceted process, transforming health services globally [65]. At the same time, there are increasing incidence and prevalence of chronic conditions, which already is represented in one-third of people aged 16 and over in the OECD countries [66]. Additionally, the extensive global challenge in treatment adherence in chronic diseases with average rates about 50% [67]. Given these factors, the authors are convinced this study has great transferability with findings of high relevance to other current issues. However, that does not preclude the acknowledgement of some study findings as valid in limited circumstances. The Nordic countries have one of the world’s highest incidences of IBD [68]. In a global setting, no more than roughly seven million people have been reported to live with IBD [69], whereas three millions of these live in Europe [16]. A large share of the world’s IBD population also live in the United States [70]. However, the transferability of these findings may still be limited considering differences in social context, such as the distinction in the public funding of healthcare services in the Nordic countries compared to the United States [71].

TA is a flexible qualitative analysis strategy that is compatible with different paradigms, given that the theoretical position, epistemological assumptions and reflexive aspects of the approach are all accounted for [54]. We have strived to the best of our ability for a transparent presentation of the results, facilitating for the reader to assess our interpretation and data analysis. Considering reflexivity, we identified preliminary assumptions, perspectives, background and motives for critical self-reflection [63], and the findings were discussed among all authors as a measure to provide internal and external perspectives of the setting. A reflexive journal was also maintained throughout the study.

Basic content analysis is limited in its assumption that the words that are studied have clear and precise meanings independent of the time of the analysis and the interpreter’s location and culture [72, p.29]. Additionally, the EHR notes are the result of what the individual nurse considered important for further continuity in patient treatment and service performance. Consequently, there is a possibility that patients have discussed other topics during telephone conversations that were not considered important and therefore not documented. The results are subsequently insufficient for conclusively predicting what different patient groups need to know the most.

In addition, the skewed marginals within the category of ‘other reasons’ may have contributed to an inadequate kappa (κ = .47) [73, p.482]. However, the category remained included to illustrate what topics other than symptoms, medicines and examinations and tests IBD patients called in to the hospital to discuss.

## Conclusion

The aim of this study was to identify key aspects required for digital promotion of self-management in IBD by eliciting IBD patients’ health information needs and identifying the preferences of HCP and IBD patients in relation to the digital provision of health services in IBD. This study has revealed important insights for developing and facilitating a digital health information service. Based on the findings from the review of EHR summaries from the IBD help line, patients appeared in general to request information about symptoms, medical treatment and results from faecal tests, endoscopic examinations and blood samples. The interviews provided further insights into the health informational needs of IBD patients, shifting the focus to information that is more relevant for patients’ daily self-management of the disease, such as their own health data, general disease and treatment information, and practical information that promotes the management and prevention of symptoms. The interviewed HCPs emphasised the importance of not increasing their workload, to ensure the continuance of the quality of care currently provided by healthcare services; they also suggested potential challenges that should be considered when making test results available to patients, especially in relation to results from blood samples. Taken together, the findings delineate key aspects of developing and adapting an accessible, convenient and usable digital health information service.

This study provided findings that must be considered when developing, adapting and implementing a digital health information service for patients with IBD. To facilitate the process, additional studies would be beneficial that provide more knowledge about the association between health information needs and IBD patients’ disease and conditions outside of the clinical setting, as well as their ability to actively use digital health information to promote treatment adherence and ensure optimal health.

### Electronic supplementary material

Below is the link to the electronic supplementary material.

Supplementary Material 1

Supplementary Material 2

## Data Availability

The datasets generated and analysed during the current study are not publicly available due to ethical agreements related to the privacy of the individuals who participated in the study. The corresponding author may be contacted to explore data sharing options.

## Abbreviations

CD: Crohn’s disease
EHR: electronic health record
HCP: healthcare professional
IBD: inflammatory bowel disease
NAV: Norwegian Labour and Welfare Administration
REC Southeast: Southeast Regional Committee for Medical Research Ethics
RN: registered nurse
TA: thematic analysis
UC: ulcerative colitis
