# Supplementary material for: Exploring the foundations of a digital health information service for patients with inflammatory bowel disease: a mixed method study in Gravitate-Health
Source: BMC Gastroenterol. 2024 May 24;24:184. doi: 10.1186/s12876-024-03272-1 (PMC11127442; doi:10.1186/s12876-024-03272-1)
Supplement: Supplementary file 1 — Supplementary Material 1 [file 12876_2024_3272_MOESM1_ESM.docx]

Interview guide: Needs for health information in digital solutions for self-management, adapted to patients’ disease activity and diagnosis.

Patients

| Introduction | | |
| --- | --- | --- |
| **Present interviewer** | |  |
| **Presentation of interviewee** | Can you tell me a little bit about yourself? E.g., name, age, diagnosis, etc. |  |
| **Explanation of the purpose of the interview** | Target group: Patients with inflammatory bowel disease |  |
|  | Purpose: The interview aims to gain insight into the needs for health information in digital solutions for patients with IBD. The focus of the interview will be to explore barriers and facilitators of health information and health literacy, and to facilitate the development of a digital healthcare service considering individuals’ disease, health challenges, and information needs. The result from these interviews intends to be published in a research article. The results will be relevant for the design of the digital platform to be tested later. It is expected that this interview will take approximately one hour to complete. Participation in the interview is voluntary. You can withdraw from the interview at any time and without providing a reason. Withdrawing will have no negative consequences for you or your treatment if you choose to do so. If you withdraw, your information will not be further used for research purposes. |  |
| **Start recording the interview in accordance with the declaration of consent. Remind participant that the recordings will be deleted after the results are published.** | |  |
| Interview | | |
| Topic | Key question(s) |  |
| **Sources of information for self-management and treatment**  Knowledge and facilitation of information about IBD to live well with the condition. | When you seek information related to IBD, where do you typically look for this information today? E.g., healthcare system, patient associations, other patients, internet, mobile applications, other sources, etc. |  |
| **Content and availability of health information** | What is the content of this information? |  |
| **Health information needs in the treatment of IBD**  Inflammatory bowel disease can manifest in various ways. The treatment of inflammatory bowel disease can therefore involve multiple components and often requires customization and adaptation for everyone. | What is it that you have wondered and are wondering about in the treatment of your own illness? E.g. diagnosis, medications, other therapy, examinations, tests, etc. |  |
| **Health information needs in the self-management of IBD**  Healthcare can provide treatment for patients in their encounters. However, IBD is a condition that is present for the individual even when they are not in contact with the healthcare system. | How accessible/comprehensible do you find the information you desire to be? E.g. language, relevance, etc. |  |
|  | Are there any specific things you do in your daily life to manage your illness and symptoms? E.g. diagnosis, medications, therapy, examinations, tests, disease progression, symptoms, other things, etc. |  |
| **We are approaching the end of the interview. There is one point left before we conclude.** | |  |
| **Desires and needs for future services.** | This research project aims to facilitate a digital service that provides access and understanding of health information and healthcare services that promote patient self-management of their own illness.  What would you like such a service to include? |  |
| End | | |
| **In this interview, I have asked you about the needs for health information in digital solutions for patients with IBD. We have now covered the topics I wanted to address.** | | |
| **Moderator provides an oral summary of the interview** | Sources of information for self-management and treatment |  |
|  | Content and availability of health information |  |
|  | Health information needs in the treatment of IBD |  |
|  | Health information needs in the self-management of IBD |  |
|  | Desires and needs for future services |  |
| **Conclusion of the interview** | Is this a reasonable interpretation of what has been said? |  |
|  | Have I understood you correctly? |  |
|  | Is there anything you are curious about or would like to bring up that has not been mentioned? |  |
| **Thank you very much for participating in this interview!** | |  |
