# Supplementary material for: Exploring the foundations of a digital health information service for patients with inflammatory bowel disease: a mixed method study in Gravitate-Health
Source: BMC Gastroenterol. 2024 May 24;24:184. doi: 10.1186/s12876-024-03272-1 (PMC11127442; doi:10.1186/s12876-024-03272-1)
Supplement: Supplementary file 2 — Supplementary Material 2 [file 12876_2024_3272_MOESM2_ESM.docx]

Interview guide: Perspectives related to the delivery of digital services for self-management, adapted to patients’ disease activity and diagnosis.

Healthcare Professionals

| Introduction | | |
| --- | --- | --- |
| **Present moderator and secretary** | |  |
| **Presentation of interviewees** | Can you tell us a little about who you are? E.g., name, age, profession, education, etc. |  |
| **Explanation of the purpose of the interview** | Target group: Patients with inflammatory bowel disease |  |
|  | Purpose: The focus group interviews are intended to provide insight into the needs for health information in digital solutions for patients with inflammatory bowel disease. The focus of the interviews will be to shed light on barriers and promoters of health information and health skills and facilitate the development of a digital health service with regard to the individual patient's illness, health challenges and information needs. The results from these interviews will be published in a research article. The results will be important for the design of the digital platform which will then be tested. It is expected that this interview will take approx. one hour to complete. Participation in the interview is voluntary. You can withdraw from the interview at any time and without giving any reason. There will be no negative consequences for you if you choose to withdraw. If you withdraw, your information will not be further researched. |  |
| **Start recording the interview in accordance with the declaration of consent. Remind participants that recordings will be deleted after the results are published.** | |  |
| Interview | | |
| Topic | Key questions | |
| **Associations**  The overall theme of this interview is digital health information and healthcare services for patients with inflammatory bowel disease. | What do you prioritize/consider that patients need to know about? |  |
| **Health information needs in the treatment of IBD**  Inflammatory bowel disease can manifest in various ways. The treatment of inflammatory bowel disease can therefore involve multiple components and often requires customization and adaptation for everyone. | What do you perceive to be the most common concerns patients have regarding the treatment of their illness. What are the most frequently asked questions? E.g., diagnosis, medicines, therapy, examinations, other topics, etc. |  |
| **Health information needs in the self-management of IBD**  Healthcare can provide treatment for patients in their encounters. However, IBD is a condition that is present for the individual even when they are not in contact with the healthcare system. | What kind of information about IBD do you believe is necessary to promote patients’ self-management in their everyday lives?  What are the things that patients can do more independently than they currently do? E.g., medicines, other therapy, examinations, disease progression, symptoms, other, etc. |  |
| **Sources of information for self-management and treatment** | Information about IBD can be crucial for living well with the condition. When patients seek information related to IBD, where do they access this information (healthcare system, patient associations, other patients, the internet, mobile applications, other sources)?  What do you understand by the term ‘health literacy’?   - In what ways do you perceive that patient information meets the patients’ health literacy? - Do you believe patients perceive the information as accessible (language, relevance, etc.)? - Would it be useful to tailor information based on patients’ health literacy? If so, how should this be considered? How can this be done today? Example? |  |
| **We are approaching the end of the interview. There are two points left before we conclude.** | | |
| **Desires and needs for future services.**  This research project aims to develop a digital service that facilitates access to and understanding of health information and healthcare services, promoting IBD patients’ self-management of their condition. | What challenges are there with digital services from a clinical perspective? |  |
|  | What do you believe such a service should include? |  |
| End | | |
| **In this interview, we have asked you about the needs for health information in digital solutions for patients with inflammatory bowel disease. We have now covered the topics we wanted to address.** | | |
| **Moderator provides an oral summary of the interview** | Associations |  |
|  | Health information needs in the treatment of IBD |  |
|  | Health information needs in the self-management of IBD |  |
|  | Sources of information for self-management and treatment |  |
|  | Desires and needs for future services. |  |
| **Conclusion of the interview** | Is this a reasonable interpretation of what has been said? |  |
|  | Have we understood you correctly? |  |
|  | Is there anything you are curious about or would like to bring up that has not been mentioned? |  |
| **Thank you very much for participating in this interview!** | |  |
